# Advanced Fusion Imaging and Contrast-Enhanced Imaging (CT/MRI–CEUS) in Oncology

**DOI:** 10.3390/cancers12102821

**Published:** 2020-09-30

**Authors:** Vincent Schwarze, Johannes Rübenthaler, Constantin Marschner, Matthias Philipp Fabritius, Johannes Rueckel, Nicola Fink, Daniel Puhr-Westerheide, Eva Gresser, Matthias Frank Froelich, Moritz Ludwig Schnitzer, Nils Große Hokamp, Saif Afat, Michael Staehler, Thomas Geyer, Dirk-André Clevert

**Affiliations:** 1 Department of Radiology, University Hospital LMU, Marchioninistrasse 15, 81377 Munich, Germany; Johannes.Ruebenthaler@med.uni-muenchen.de (J.R.); constantin.marschner@med.uni-muenchen.de (C.M.); Matthias.Fabritius@med.uni-muenchen.de (M.P.F.); johannes.rueckel@med.uni-muenchen.de (J.R.); nicola.fink@med.uni-muenchen.de (N.F.); daniel.puhr-westerheide@med.uni-muenchen.de (D.P.-W.); eva.gresser@med.uni-muenchen.de (E.G.); moritz.schnitzer14@gmail.com (M.L.S.); thomas.geyer@med.uni-muenchen.de (T.G.); dirk.clevert@med.uni-muenchen.de (D.-A.C.); 2 Institute of Clinical Radiology and Nuclear Medicine, University Medical Center Mannheim, Theodor-Kutzer-Ufer 1-3, 68167 Mannheim, Germany; Matthias.Froelich@medma.uni-heidelberg.de; 3 Institute for Diagnostic and Interventional Radiology, Faculty of Medicine and University Hospital Cologne, University Cologne, Kerpener Str. 62, 50937 Cologne, Germany; nils.grosse-hokamp@uk-koeln.de; 4 Department for Diagnostic and Interventional Radiology, Eberhard Karls University Tuebingen, University Hospital Tuebingen, Hoppe-Seyler-Str. 3, 72076 Tuebingen, Germany; saif.afat@med.uni-tuebingen.de; 5 Department of Urology, University Hospital LMU, Marchioninistrasse 15, 81377 Munich, Germany; michael.staehler@med.uni-muenchen.de

**Keywords:** fusion imaging, ultrasound, CEUS, CT, MRI, kidney, RCC, liver, HCC, oncology

## Abstract

**Simple Summary:**

Fusion imaging depicts an innovative technique by which previously performed computed tomography/magnetic resonance imaging can be integrated and reconstructed with advanced contrast-enhanced ultrasound using modern ultrasound devices in a real-time manner. Fusion imaging allows for complementing strengths and reducing restrictions of the combined imaging modalities. The visualization of parenchymal and tumoral microperfusion by contrast-enhanced ultrasound can be dynamically fused and assessed with images from previous cross-sectional studies and may help to decipher underlying entities of indeterminate lesions or validate suspicious morphology. The findings from our study demonstrate the benefits of fusion imaging for evaluating focal hepatic and renal lesions. The excellent safety profile, accessibility, repeatability and cost-effectiveness are advantages of fusion imaging which make it a powerful diagnostic tool for the modern radiologist.

**Abstract:**

Fusion imaging depicts an innovative technique that facilitates combining assets and reducing restrictions of advanced ultrasound and cross-sectional imaging. The purpose of the present retrospective study was to evaluate the role of fusion imaging for assessing hepatic and renal lesions. Between 02/2011–08/2020, 92 patients in total were included in the study, of which 32 patients had hepatic lesions, 60 patients had renal lesions. Fusion imaging was technically successful in all patients. No adverse side effects upon intravenous (i.v.) application of *SonoVue^®^* (*Bracco*, Milan, Italy) were registered. Fusion imaging could clarify all 11 (100%) initially as indeterminate described hepatic lesions by computed tomography/magnetic resonance imaging (CT/MRI). Moreover, 5/14 (36%) initially suspicious hepatic lesions could be validated by fusion imaging, whereas in 8/14 (57%), malignant morphology was disproved. Moreover, fusion imaging allowed for the clarification of 29/30 (97%) renal lesions initially characterized as suspicious by CT/MRI, of which 19/30 (63%) underwent renal surgery, histopathology revealed malignancy in 16/19 (84%), and benignity in 3/19 (16%). Indeterminate findings could be elucidated by fusion imaging in 20/20 (100%) renal lesions. Its accessibility and repeatability, even during pregnancy and in childhood, its cost-effectiveness, and its excellent safety profile, make fusion imaging a promising instrument for the thorough evaluation of hepatic and renal lesions in the future.

## 1. Introduction

Conventional ultrasound, comprising native B-mode and Color Doppler, is frequently applied as an imaging modality for initial abdominal investigation, including kidney and liver imaging [1,2,3]. Ultrasound is applied as a screening tool in patients with chronic diseases, who thus are predisposed to developing cancer, e.g., renal cell carcinoma (RCC) or hepatocellular carcinoma (HCC) [4,5]. It is also used as the imaging instrument of choice when patients present with acute abdominal symptoms. Image acquisition by ultrasound is based on scattering, reflecting, and frequency shifting of ultrasound waves by different tissues. Due to the physics behind imaging acquisition by ultrasound, obesity, a limited acoustic window, or bowel gas depict shortcomings of conventional ultrasound. The administration of intravenous microbubbles for enabling vascular and parenchymal contrast enhancement allows for improved visualization of abdominal pathologies, e.g., demarcate focal liver lesion surrounded by steatotic liver parenchyma [6]. The advantages of contrast-enhanced ultrasound (CEUS) are that it can immediately and repeatably be applied, its cost-effectiveness, and its excellent non-ionizing safety profile [7]. Nevertheless, CEUS cannot overcome all shortcomings of conventional ultrasound. Often, cross-sectional imaging modalities, such as computed tomography (CT) and magnetic resonance imaging (MRI), are critical and inevitable, especially under acute or traumatic circumstances of the patient. Thus, CT or MRI is recommended by the American College of Radiology (ACR) for assessing certain acute abdominal situations [8,9,10]. Focal liver or renal lesions are frequently and incidentally registered due to the increasing use of elaborate CT and MRI scans. Because CT and MRI examinations often are performed without adequate protocols that allow for specific scrutinization of the incidentally found parenchymal lesions, their underlying entities often remain indeterminate, and further diagnostic evaluation is necessary [11]. A thorough evaluation is mandatory before CT and MRI are re-done with optimized protocols. Ionizing radiation, in case of CT, potential renal affection due to iodinated or gadolinium-based contrast agents, potential allergic predisposition to contrast agents, as well as the relevant financial costs, must be considered. 

Modern high-end ultrasound devices allow for the integration and adequate reconstruction of previously performed CT or MRI scans, thereby enabling simultaneous and real-time computerized fusion of former cross-sectional studies with live ultrasound images in the same and additional planes [12,13]. Fusion imaging depicts an innovative technique by which the assets of combined imaging modalities, such as comprehensive field of view and high-contrast resolution of CT/MRI, and high spatial resolution of ultrasound in real-time, can be complemented and restrictions can be minimized, e.g., the limited acoustic window of ultrasound is extended by the wide field of view of CT and MRI [14]. Of note, fusion imaging can be conducted with native B-mode, Color Doppler, CEUS, and elastography, which facilitates thorough and dynamic scrutiny of focal parenchymal lesions of interest [15]. The visualization of tissue and tumoral microperfusion by CEUS can be dynamically fused and analyzed with images from previous cross-sectional studies, thereby further elevating the confidence of the observer. Of note, compared to elaborate cross-sectional imaging modalities, CEUS and advanced fusion imaging are easily accessible and repeatable, comparably inexpensive, and have an excellent safety profile [7]. By using fusion imaging, the ultrasound examination depends less on the capability of the observer to mentally fuse findings from previous CT/MRI scans with recent sonographic findings. The advantage of using fusion imaging to ablate liver lesions and to monitor subsequent therapeutic outcome was already described [16,17,18]. Furthermore, fusion imaging of CEUS and CT was shown to help the placement of endovascular aortic repair (EVAR) and further improve the visualization of graft endoleaks [19,20]. 

The purpose of the present study was to evaluate the role of advanced fusion imaging for assessing liver and renal lesions at our specialized University Hospital. 

## 2. Results

Between 02/2011 and 08/2020, 32 patients with focal liver lesions and 60 patients with focal renal lesions underwent fusion imaging (either CT/CEUS or MRI/CEUS). 

The mean age of the included patients with focal liver lesions was 54 years (range: 17–81 years) with a female predominance (female:male-ratio = 22:8, approximately 2.8:1). The mean size of focal liver lesions was 1.9 cm (range: 0.5–6.2 cm). Table S1 gives a detailed description of the included patients with focal liver lesions. 

Of all 11 focal liver lesions that were initially described as indeterminate in either CT or MRI, fusion imaging elucidated the underlying entity: 4/11 (36%) uncomplicated liver cysts, 3/11 hemangioma (27%), 2/11 focal nodular hyperplasia (FNH) (18%), 1/11 hemorrhagic liver cyst (9%), and 1/11 (9%) vascular pseudolesion. 

Fusion imaging validated malignancy in 5/14 (36%) focal liver lesions that were previously reported to show suspicious morphology in either CT or MRI, with a mean size of 1.9 cm (range: 1.0–3.7 cm). In patient #12, morphological findings from both CT and fusion imaging implicated liver metastases, which were histopathologically confirmed as such by underlying rectal carcinoma. The suspicious liver lesion in patient #15 turned out to be a cholangiocellular carcinoma (CCC) after left hemihepatectomy and histopathological scrutiny. Liver metastases from breast cancer were histopathologically revealed in patient #26, in whom CT and fusion imaging indicated malignant liver lesions. After patient #27 underwent left hemihepatectomy, histopathology revealed HCC and, thus, confirmed suspicious findings from MRI and fusion imaging. Findings from MRI and fusion imaging strongly suggested HCC in patient #31. Due to advanced age and limiting comorbidities, no surgical treatment/biopsy nor histopathological analysis was conducted. 

Findings from fusion imaging clarified underlying entities in the remaining 8/14 (57%) focal liver lesions that were initially described suspicious and had a mean size of 1.3 cm (range: 0.7–4.5 cm): 4/14 (29%) hemangioma, 2/14 (14%) FNH, 1/14 (7%) uncomplicated liver cyst, 1/14 (7%) no malignancy. In 1/14 (7%), no specific correlation could be achieved by fusion imaging. There was no significant difference in lesional diameter between malignant and benign focal liver lesions (5/14 vs. 8/14, *p* = 0.55), all of which were initially described as suspicious by CT or MRI.

In patient #14, fusion imaging did not reveal any other morphological information besides priorly described calcification by CT. Intrahepatic abscess formation, as registered in MRI, was validated by fusion imaging in patient #17. Furthermore, fusion imaging could visualize successful radiofrequency ablation (RFA) of a single hepatic metastasis of ovarian carcinoma in patient #21. A complicated liver cyst was reported in patient #18 by MRI, which was further evaluated as a hemorrhagic liver cyst by fusion imaging. 

Of note, in 6/32 (19%) patients, findings from CT/MRI could not be correlated in a conventional ultrasound, and a further 4/6 (67%) could not be correlated due to massive steatosis hepatis. Instead, fusion imaging allowed for precise correlation. 

With fusion imaging being diagnostic reference, cross-sectional imaging (CT/MRI) showed a pooled sensitivity of 80%, specificity of 67%, positive predictive value (PPV) of 31%, and negative predictive value (NPV) of 95% for evaluating liver lesions in our cohort. 

**Figure 1:**
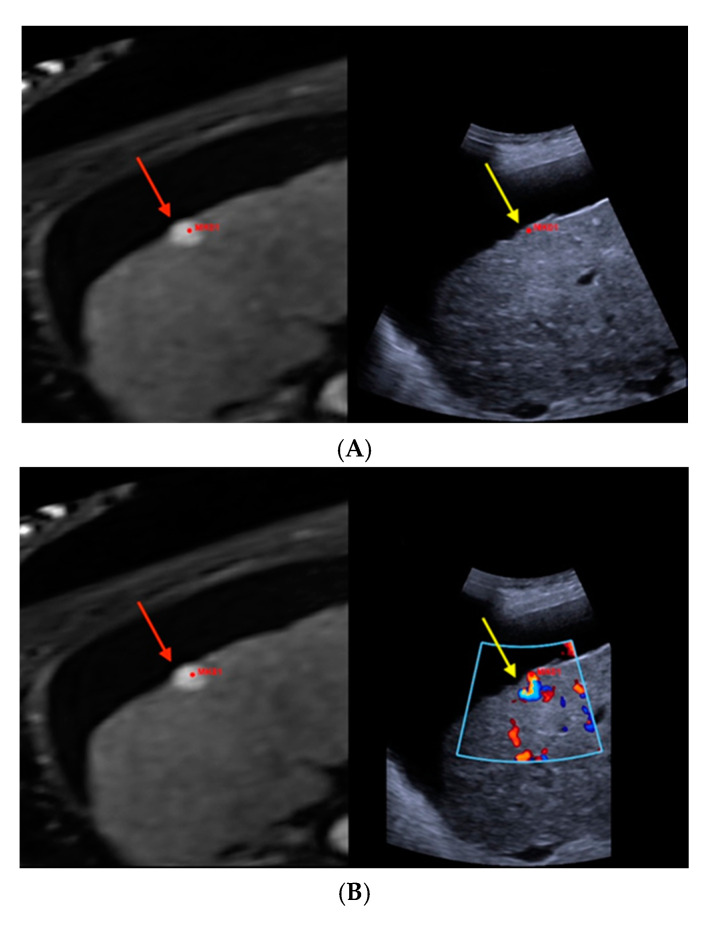

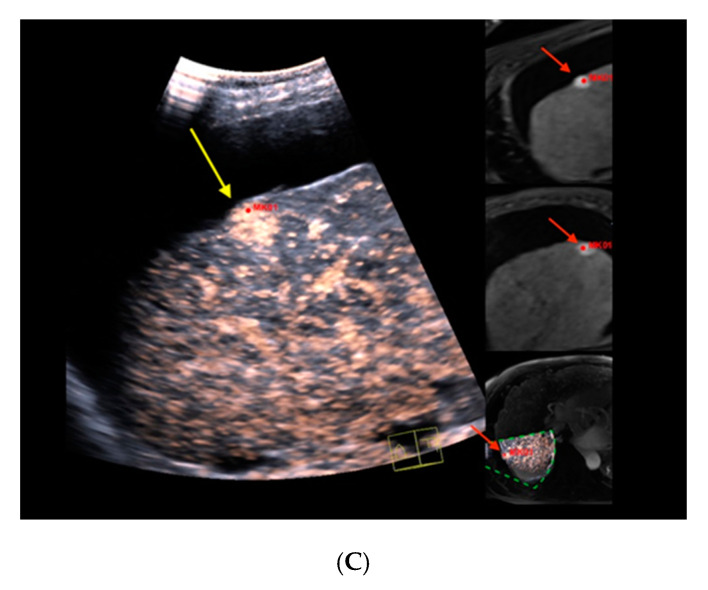
Real-time magnetic resonance imaging-/contrast-enhanced ultrasound (MRI-/CEUS)-fusion imaging of a hypervascularized focal liver lesion in a cirrhotic patient. (**A**) A hypervascularized subcapsular focal lesion in liver segment 7 was registered in MRI, arterial phase (left), a target lesion (MK01, red) was placed for precise correlation with native B-mode (right) in a side-by-side manner. The focal lesion otherwise was barely detectable by conventional ultrasound. (**B**) Additional Color Doppler showed livid hypervascularization of the lesion (right), a corresponding plane of MRI, arterial phase (left). (**C**) Peripheral-to-central contrast-enhancement and, finally, homogeneous contrast enhancement was registered in CEUS (left, maximized), implicating focal nodular hyperplasia. The software interface of the ultrasound device showed four different images: real-time CEUS images (left, maximized), MRI datasets in axial (upper right) and sagittal (middle right) reformation, and a real-time 3D navigation of the MRI-/CEUS-fusion imaging (lower right), (lesion marked by red arrows).

Figure 1 illustrates the MR-morphological correlate of a suspiciously hypervascularized focal liver lesion in a cirrhotic patient, which fusion imaging unraveled as underlying FNH.

An overview of findings from cross-sectional imaging (CT/MRI) and corresponding correlates by fusion imaging of the included focal liver lesions are illustrated in Table 1.

The mean age of the included patients with focal renal lesions was 64 years (range: 31–87 years) with a male predominance (male: female-ratio: 41:19, approximately 2.2:1) (Table 2). The mean size of focal renal lesions was 2.4 cm (range: 0.7–10.0 cm). The focal renal lesions were localized in a right:left-ratio = 31:29, approximately, 1.1:1). Table S2 depicts a detailed description of the included patients with focal renal lesions. The mean size of the analyzed focal renal lesions was 2.5 cm (range: 0.7–10.0 cm). 

Half of the analyzed focal renal lesions, 30/60 (50%), were initially described as suspicious by CT or MRI, which were subsequently evaluated by fusion imaging as follows: 4/30 (13%) as Bosniak 1 renal cysts with a mean size of 2.0 cm (range: 1.0–2.5 cm), 2/30 (7%) as Bosniak 2 renal cysts with a mean size of 0.9 cm (range: 0.8–1.0 cm), 3/30 (10%) as Bosniak 2F renal cysts with a mean size of 3.7 cm (range: 0.7–7.5 cm), 6/30 (20%) as Bosniak 3 renal cysts with a mean size of 1.9 cm (range: 1.0–3.0 cm), 13/30 (43%) as Bosniak 4 renal cysts with a mean size of 2.6 cm (range: 0.8–5.0 cm), 1/30 (3%) as angiomyolipoma with a size of 3.5 cm (pat. #17), 1/30 (3%) no specific correlation with a lesional diameter of 1.0 cm registered in CT (pat. #7). Patient #35 underwent right partial nephrectomy in whom 1.0 cm suspicious renal lesion was described in MRI and was subsequently assessed as a Bosniak 2 renal lesion by fusion imaging; histopathology revealed angiomyolipoma. Patient #23 underwent right partial nephrectomy, and histopathology revealed papillary RCC upon report of a suspicious renal lesion in MRI and categorized as a Bosniak 2F renal lesion by fusion imaging due to discrete intracystic septations and lack of contrast-enhancement. Five out of six (83%) patients in whom fusion imaging described Bosniak 3 renal lesions after initial cross-sectional imaging showed suspicious lesions, underwent (partial) nephrectomy. Histopathology, finally, revealed renal oncocytoma in 2/5 (40%and, papillary/chromophobe/clear-cell RCC in 1/5 (20%), respectively. Ten out of twelve patients in whom fusion imaging evaluated a Bosniak 4 renal lesion after conspicuous cross-sectional imaging underwent (partial) nephrectomy. Histopathology confirmed underlying clear-cell RCC in 7/10 (70%), papillary RCC in 2/10 (20%), and chromophobe RCC in 1/10 (10%). 

Initial evaluation, either by CT or MRI, of renal lesions, remained indeterminate in 20/60 (33%) of the included patients in whom assessment by fusion imaging was conducted afterward as follows: 8/20 (40%) as Bosniak 2F renal lesions with a mean size of 2.7 cm (range: 1.1–4.5 cm), 6/20 (30%) as Bosniak 1 renal lesions with a mean size of 1.3 cm (range: 0.7–2.0 cm), 1/20 (5%) as Bosniak 3 renal lesion with a size of 1.2 cm, 1/20 (5%) as a Bosniak 2 renal lesion with a size of 2.1 cm, 1/20 (5%) as an angiomyolipoma with a size of 1.0 cm, 1/20 (5%) as a renal infarction with a diameter of 1.3 cm, 1/20 (5%) as a parenchymal alteration in the context of pyelonephritis, mean diameter of 1.5 cm, 1/20 (5%) as an indeterminate, benign lesion, which was histopathologically confirmed as an angiomyolipoma. Three focal renal lesions were initially described as uncomplicated cysts by CT/MRI; 1/3 was upgraded to a Bosniak 2F (pat. #53), 1/3 was upgraded to a Bosniak 3 (pat. #39), and 1/3 was confirmed as a Bosniak 1 (Pat. #5) by fusion imaging, all of which were not histopathologically correlated. Two focal renal lesions were attributed to the Bosniak 2 category by cross-sectional imaging. Upgrading to Bosniak 2F (Pat. #13) and downgrading to Bosniak 1 (pat. #38) was done by fusion imaging. Moreover, validation of renal hematoma and exclusion of active bleeding due to derailed oral anticoagulation (warfarin) was accomplished by fusion imaging in (pat. #18). In patient #24, the findings from MRI suspected a recurrence of clear-cell RCC, which was verified by fusion imaging. Non-enhancing parenchymal defect after Cyberknife treatment of clear-cell RCC in patient #25 was registered in MRI-CEUS-fusion imaging. An angiomyolipoma of the left kidney measuring 1.4 cm was suspected in an unenhanced MRI in a pregnant patient at 26 weeks of pregnancy (pat. #60). Corresponding MRI-CEUS-fusion imaging showed a hypoechoic and hypervascularized sonomorphological correlate, which after intravenous (i.v.) application of *SonoVue^®^*, showed early arterial enhancement and delayed wash-out. A subsequent ultrasound-guided biopsy and histopathological analysis, finally, validated an underlying angiomyolipoma.

With fusion imaging being diagnostic reference, cross-sectional imaging (CT/MRI) showed a pooled sensitivity of 91%, specificity of 68%, positive predictive value (PPV) of 80%, and negative predictive value (NPV) of 85% for assessing renal lesions in our cohort. 

**Figure 2:**
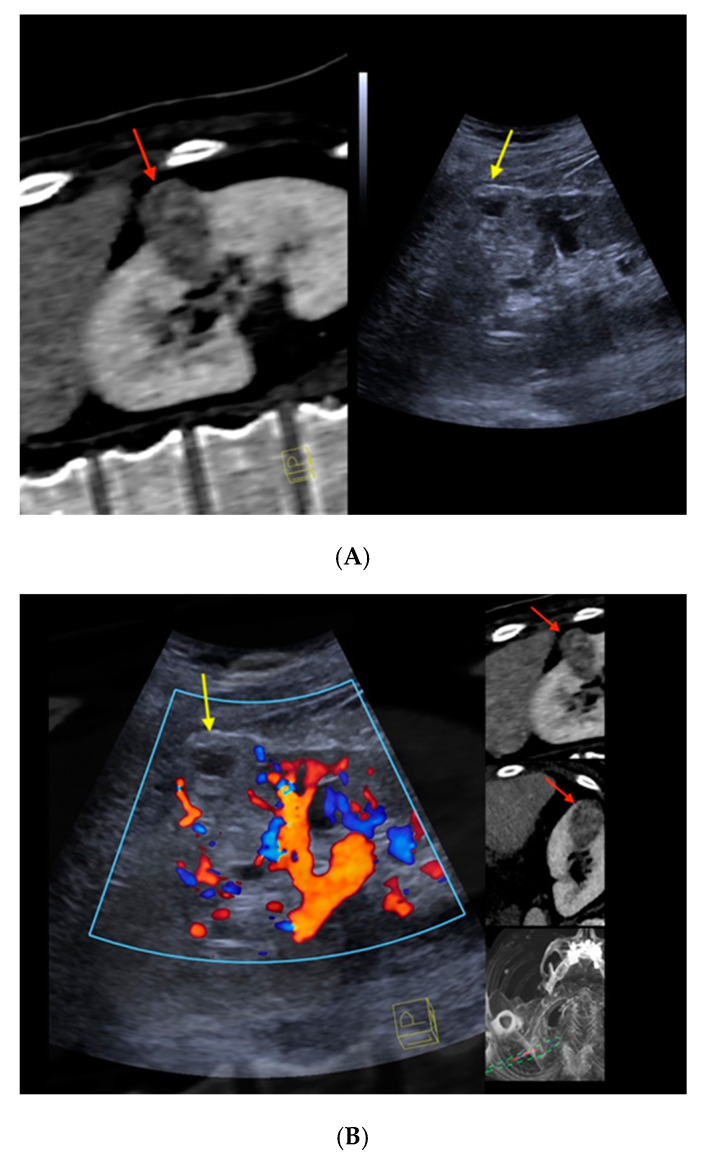

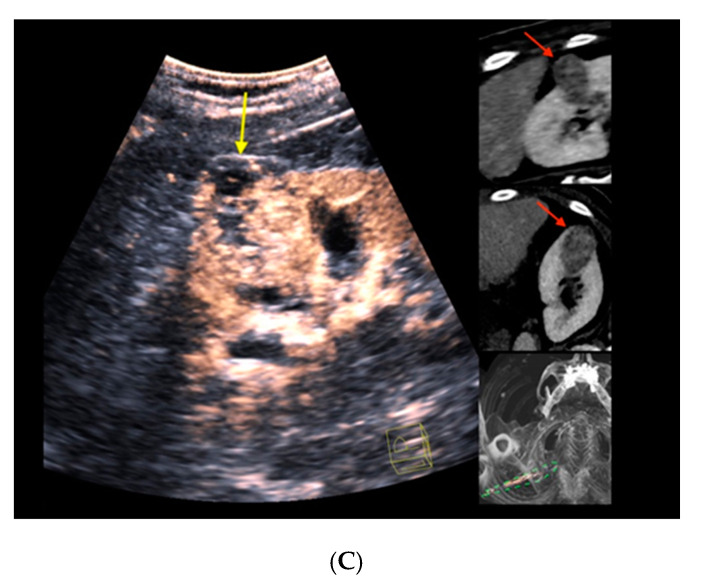
Real-time CT-/CEUS-fusion imaging of a complex renal cystic lesion. (**A**) Known complex renal cystic lesion with indicated septations and solid components in the right kidney from previous contrast-enhanced CT (left, red arrow) is displayed in a side-by-side mode with native B-mode (right, yellow arrow) by a high-end ultrasound system. (**B**) Additional Color Doppler did not reveal hypervascularization of the lesion (yellow arrow). The software interface of the ultrasound device showed four different images: the real-time Color Doppler image (left, maximized), the CT imaging dataset in sagittal (upper right) and axial (middle right) reformation (lesion marked by red arrows), and a real-time 3D navigation of the Fusion Imaging (lower right). (**C**) Contrast-enhanced ultrasound allowed for visualization of early arterial microperfusion of solid components of the lesion, implicating malignancy (left, maximized), (lesion marked by red arrows in corresponding CT images). The patient underwent partial nephrectomy. Histopathology, finally, revealed underlying clear-cell renal cell carcinoma.

Figure 2 depicts the morphological correlates from CT/CEUS-fusion imaging of a clear-cell RCC. An overview of the findings from cross-sectional imaging (CT/MRI) and corresponding correlates by fusion imaging of the included renal liver lesions is depicted in Table 2.

## 3. Discussion

Beyond doubt, conventional ultrasound remains a mainstay for the initial evaluation of acute abdominal symptoms [2,3]. In addition, ultrasound is recommended as the primary imaging modality in the context of screening patients with chronic liver and kidney diseases, who thus are prone to cancer development [5,21]. Analyzing the contrast dynamics, in particular, arterial phase-contrast enhancement and venous wash-out of focal liver lesions may allow for the determination of the underlying entity [4]. In terms of assessing renal lesions, relevant morphological features that allow for the discrimination between benign and malignant origin comprise septations, nodular components, mural thickening, calcifications, and contrast-enhancement [22]. Since these morphological features of liver and renal lesions may be very discrete, imaging modalities with high spatial and temporal resolutions are necessary for visualization. Fusion imaging facilitates the combination of dynamic CEUS with CT/MRI, thereby exploiting the advantages and reducing the restrictions of both imaging modalities [12]. The purpose of the present study was to evaluate fusion imaging for assessing liver and kidney lesions at our specialized University Hospital. 

Hepatocellular carcinoma (HCC) depicts the most frequent primary hepatic cancer, the sixth most common cancer with the second-highest tumor mortality [23,24]. The imaging modality of choice for liver cancer surveillance is ultrasound, which showed diagnostic sensitivities up to 80% and specificities of > 90% [25]. Semiannual liver ultrasound screenings are recommended by the leading societies [4,26]. The Liver Imaging Reporting and Data System (LI-RADS) facilitates standardized screening, surveillance, and treatment response evaluation of HCC by CT, MRI, or CEUS [27]. Malignant liver lesions characteristically feature a modified vascularization pattern juxtaposed to non-tumorous liver tissue, predominantly arterial vs. portal venous supply, respectively, resulting in arterial hyperenhancement and venous hypoenhancement/wash-out in contrast-enhanced CT, MRI, or CEUS [28]. Many clinical trials already described the high diagnostic accuracy of CEUS for analyzing focal liver lesions [29,30,31]. A recent prospective study demonstrated comparably accurate findings in CEUS versus CT in terms of detecting hypervascularization of HCC. Of note, the study showed a superior diagnostic performance of CEUS for visualizing venous wash-out of HCC [32]. The results from a multicenter study could show an equivalent diagnostic accuracy of CEUS versus CT in terms of liver tumor differentiation and specification [29]. The diagnostic superiority of CEUS compared to more elaborate cross-sectional imaging modalities for smaller HCC lesions was previously described. A retrospective study found out that typical morphological HCC-features were less frequently registered by MRI than visualized by CEUS [33]. Furthermore, the cost-effectiveness of CEUS in the context of HCC surveillance was previously described [34]. Nonetheless, CEUS still is not recommended as the primary imaging modality by the leading societies [4,26,35,36] but stated as an adjunct secondary imaging modality. Up to date, fusion imaging has not been recommended by the leading societies for diagnostic workup and follow-up of hepatic lesions. The World Federation for Ultrasound in Medicine and Biology (WFUMB) states that fusion imaging is a pivotal tool for interventional tumor ablation [37]. 

Our findings showed that fusion imaging allowed for accurate correlation of liver lesions between CT/MRI and CEUS in 31/32 (97%) patients. Eleven out of thirty-two (34%) liver lesions that were initially categorized as indeterminate by CT/MRI could be elucidated as of benign origin by fusion imaging; thus, avoiding further (invasive) diagnostic approaches. Moreover, fusion imaging allowed for the downgrading of 9/14 (64%) focal liver lesions that were primarily delineated as suspicious by CT/MRI. Malignant morphology was confirmed by fusion imaging in the remaining 5/14 (36%) liver lesions that already appeared suspicious at first CT/MRI. Since focal liver lesions may be located too deep in the acoustic cone or may not be visible due to their isoechogenicity compared to the adjacent liver parenchyma, they might not be registered by conventional ultrasound. Notably, accurate correlation of focal liver lesions was accomplished by fusion imaging in 6/32 (19%) patients, all of which were not detectable in conventional ultrasound, and in 4/6 (60%) due to massive steatosis hepatis. The latter findings are compatible with previous studies, which could show significantly higher detection rates by using fusion imaging compared to ultrasound/CEUS alone for visualization of focal liver lesions/HCC [38,39]. As previously described, successful treatment of a single liver metastasis by RFA could be monitored by fusion imaging, fusing pre-treatment MRI with post-treatment CEUS. Hence, a focal parenchymal defect without detectable microperfusion could be visualized [40,41,42,43]. Our results from the 32 included patients with focal liver lesions highlight the advantageous fusion of dynamic CEUS with cross-sectional imaging, either CT or MRI, minimizing limitations and utilizing strengths of both imaging modalities. 

Renal cell carcinoma (RCC) accounts for approximately 3% of all cancers [44]. In over 30% of initial diagnosis, metastases have already spread. The detection rates of RCC have considerably increased during the past decades, mainly due to progressing imaging technologies. More incidental renal lesions are frequently detected in CT and MRI scans, which were initially conducted for other reasons. Most of the incidental renal lesions are uncomplicated renal cysts [45]. Still, a relevant cohort of renal lesions remains indeterminate, among others, because of the overlapping morphologies of benign and malignant tumor entities [46]. Hence, further diagnostic assessment, biopsy, surgical (partial) resection, and histopathological analysis are of pivotal importance. The recent Bosniak classification, which was first introduced in 1986 and initially based on CT findings, allows for the stratification of renal lesions into five subtypes (1–4 plus 2F, “F” stands for “follow-up”) by which their malignant potential may be estimated; with malignancy rates as follows: Bosniak 1 and 2 almost 0%, Bosniak 2F up to 5%, Bosniak 3 and 4 up to 50% and 100%, respectively [47,48,49]. Thus, appropriate treatment of renal lesions necessitates precise categorization according to the Bosniak classification. The categorization into Bosniak subtypes is based on morphological features: thickness of the cyst wall (hairpin-thin versus thickened), septations, mural/septal calcifications, nodular components, mural/septal/solid contrast-enhancement. Contrast-enhancement can be detected either by CT/MRI or by CEUS, all three imaging modalities were previously reported to show high diagnostic accuracy in evaluating renal lesions [50,51,52,53,54,55]. Since MRI allows for remarkable soft tissue and contrast resolution, it was shown to be superior compared to CT in terms of detecting fine septations, mural and septal thickening, as well as contrast-enhancement of renal lesions. Hence, it is plausible that assessing renal lesions by MRI may result in different Bosniak stratification compared when doing so by means of CT. Computed tomography is most suitable for detecting calcification of renal lesions. Comparable diagnostic performances of CEUS compared to CT and MRI for analyzing renal lesions were previously demonstrated [56]. A meta-analysis revealed an even higher diagnostic sensitivity of CEUS compared to MRI for analyzing complex renal lesions [54]. More, it could already be shown that CEUS depicts a valid tool for clarifying incidental renal lesions detected in CT [57]. Of note, CEUS depicts a powerful tool that allows the visualization of even single microbubbles within small vessels, septations, or cystic walls [58]. Hence, precise stratification according to the Bosniak classification is feasible [59]. Up to now, the leading societies have recommended CT and MRI as the primary imaging modalities for the diagnostic work- and follow-up of complex renal lesions; some list CEUS as an adjunct imaging tool. 

Enhanced identifiability and improved evaluation of the dignity of renal lesions by using fusion imaging compared to cross-sectional imaging was already reported [60,61]. Nonetheless, fusion imaging has not been recommended by the leading societies in the context of diagnostic workup and follow-up of renal lesions so far.

Our results show that fusion imaging allowed for a feasible correlation of the renal lesions in 59/60 (98%) between CT/MRI and CEUS. In 5/60 (8%) patients, no specific correlate in conventional ultrasound (native B-mode and Color Doppler) before and after fusion imaging could be detected. Still, the renal lesions demarcated upon intravenous (i.v.) application of *SonoVue^®^* and could be scrutinized by CEUS and categorized to Bosniak subtypes: Bosniak 1 (*n* = 1), Bosniak 3 (*n* = 2), Bosniak 4 (*n* = 2). 

Fusion imaging enabled further clarification of 30/60 (50%) renal lesions which initially were characterized as suspicious. In 7/30 (23%) initially suspicious described renal lesions, no further (invasive) diagnostic approach was necessary since fusion imaging allowed for the characterization of those lesions as Bosniak 1 (*n* = 4), Bosniak 2 (*n* = 2), or angiomyolipoma (*n* = 1). In only 1/30 (3%) patient, fusion imaging was not capable of correlating the suspicious finding from prior CT. Three out of thirty (10%) renal lesions were categorized as Bosniak 2F, which, therefore, required follow-up. One patient, in whom fusion imaging could visualize a hypoechoic, septated renal lesion, measuring 3.0 cm, that featured contrast-enhancement, thus indicating Bosniak 2F, underwent partial nephrectomy 15 months later, and histopathology eventually revealed papillary RCC. Furthermore, 5/6 (83%) Bosniak 3 lesions, categorized by fusion imaging, were histopathologically verified after (partial) nephrectomy, revealing papillary, chromophobe, and clear-cell RCC each in 1/5 (20%). Benign renal oncocytoma was found in 2/5 (20%) as Bosniak 3 categorized lesions. The latter finding is compatible with a recent retrospective study that could not describe a distinct sonomorphological appearance specific for renal oncocytoma [46]. Of those patients with Bosniak 4 lesions, which were categorized as such by fusion imaging, 10/12 (83%) were histopathologically scrutinized after (partial) nephrectomy. All turned out to be malignant (clear-cell:papillary:chromophobe RCC = 7:2:1). Of note, fusion imaging allowed for the categorization of renal lesions, which were initially characterized as suspicious, to Bosniak 2F in 8/20 (40%) and thus, accentuating the relevance of follow-up of those lesions. Moreover, upgrading of renal lesions from Bosniak 1 to 2F (*n* = 1) and 3 (*n* = 1), and from Bosniak 2 to 2F (*n* = 1) was accomplished by fusion imaging. As recently described in a long-term follow-up analysis, the progression rate of Bosniak 2F lesions detected by CEUS was 7.1% within a mean time of almost 13 months [62]. The findings from the study display CEUS as a valid imaging modality for work- and follow-up of Bosniak 2F lesions. 

In addition, in one patient, the morphological appearance of a renal lesion, measuring 1.2 cm, remained indeterminate in MRI, and subsequent fusion imaging was performed. No specific correlation could be achieved by fusion imaging with conventional ultrasound upon i.v. application of *SonoVue^®^*. Arterial contrast-enhancement could, finally, be visualized, indicating the Bosniak 3 category. Due to extensive comorbidities of the patient, no further follow-up nor surgery/biopsy was undertaken. In one patient, fusion imaging validated the recurrence of RCC. Analogous to the described application of fusion imaging for monitoring treatment response upon hepatic intervention, successful Cyberknife treatment of a clear-cell RCC could be visualized by fusion imaging. Of utmost importance, fusion imaging helped strengthen findings from unenhanced MRI, thereby clarifying renal angiomyolipoma in a pregnant patient at 26 weeks of pregnancy. The patient later underwent an ultrasound-guided biopsy, and definite verification by histopathology was done. As recently demonstrated, CEUS proved a safe and reliable imaging tool to make contrast-enhanced (maternal) investigations during pregnancy feasible [63,64,65]. It could be shown that *SonoVue^®^* did not cross the placental barrier [66]. In a nutshell, our findings from 60 included patients depicted the beneficial application of fusion imaging for the precise evaluation of renal lesions.

A thorough evaluation is critical before CT and MRI examinations are performed. The ionizing radiation in the case of CT results in an elevated risk of radiation-related cancers [67,68], potential allergic predisposition against, as well as affections of the renal and thyroid function due to iodinated contrast agents, must also be considered. Limited availability, higher financial costs, restricted applicability of contrast-agents in patients with kidney failure or allergic predisposition, as well as limited usage in case of corporeal metallic medical devices, are detrimental aspects of MRI. Although possible long-term clinical effects have not been reported so far, the recently reported potential deposition of gadolinium-based contrast agents within the basal nuclei requires deliberation [69]. In sharp contrast, CEUS and its innovative integration in the context of fusion imaging are cost-effective, directly accessible, and repeatable with fewer hesitations at an excellent safety profile [7,70]. Once cross-sectional studies are performed, fusion imaging can be safely conducted in patients with renal or thyroidal disorders, allergic predispositions to iodinated or gadolinium-based contrast agents, as well as in pregnant patients and children [71]. Of importance, fusion imaging allows for the visualization of tissue and tumor microperfusion at higher temporal and spatial resolutions in a real-time manner compared to CT/MRI alone. The limited diagnostic performance of static cross-sectional imaging compared to fusion imaging as a reference modality in our cohorts emphasizes the associated diagnostic benefits of using advanced and real-time fusion imaging. The depicted advantages of fusion imaging plausibly enhance the confidence of the observer. Of course, performing fusion imaging highly depends on the skills of the observer and is susceptible to moving artifacts, e.g., due to breathing. 

Frequently oncologic patients do have relevant comorbidities, which may limit their transfer to the Radiology Department and, therefore, the use of CT or MRI, e.g., invasive ventilation or catecholamine therapy. Fusion imaging can easily be performed at the patients’ bedside, therefore, sparing potential anxiety of the patient due to delayed reporting, and the patient can immediately be informed about the findings. Doubtless, fusion imaging is not capable of replacing cross-sectional studies in terms of oncological staging.

There are some limitations to the present study. First, the patients were included retrospectively. All fusion imaging examinations were conducted by one single experienced radiologist (EFSUMB level 3). Three different up-to-date ultrasound devices were used for fusion imaging. Both cohorts of analyzed patients, with focal liver lesions and focal renal lesions, showed quite a heterogeneity with regard to lesional dignity. 

## 4. Materials and Methods

**Figure 3:**
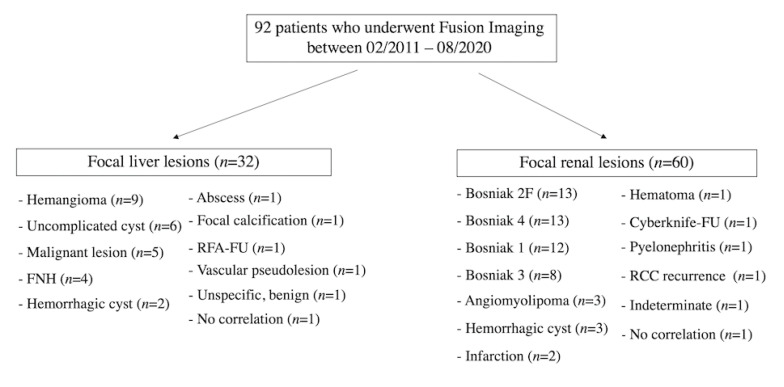
Flowchart illustrating included patients who underwent fusion imaging. FNH-focal nodular hyperplasia, RFA-FU-radiofrequency ablation-follow-up, Cyberknife-FU-Cyberknife-Follow-Up, RCC-renal cell carcinoma.

Between 02/2011–08/2020, 32 patients with focal liver lesions and 60 patients with focal renal lesions underwent fusion imaging (CT/CEUS or MRI/CEUS). In total, 92 patients underwent fusion imaging and were included in this study (Figure 3.). Two thousand, eight hundred and sixty-six liver and 2651 renal CEUS examinations were performed between 02/2011–08/2020 with fusion imaging depicting 1, 1% (32/2866) and 2, 3% (60/2651) of total CEUS examinations, respectively. Fusion imaging was technically successful in all included 92 patients. The relatively small number of included patients was due to several aspects. First, fusion imaging is not comprehensively integrated into the daily clinical routine and is not recommended by the relative leading societies yet. Moreover, knowledge of fusion imaging and its benefits, so far, have not been widespread among specialties other than Radiology. High expertise in advanced CEUS and fusion imaging is also pivotal. Lastly, in our Department of Radiology, usually, only one single skilled consultant radiologist performs CEUS/Fusion Imaging (EFSUMB Level 3).

The retrospective single-center study was approved by the local institutional ethical committee of the institutional review board (Ethics Committee, Medical Faculty, Ludwig-Maximilians-University Munich; 17-087; date of approval: 14 March 2017). All contributing authors followed the ethical guidelines for publication in *Cancers*. All study data were collected and retrieved respecting the principles expressed in the Declaration of Helsinki/Edinburgh 2002. Oral and written informed consent was obtained from all patients before advanced fusion imaging was conducted. The process of CEUS, associated risks, as well as possible complications, were thoroughly described. All advanced ultrasound examinations were conducted by one single versed consultant radiologist (EFSUMB level 3). Advanced ultrasound examination included native B-mode, Color Doppler, CEUS, and fusion imaging with a previous CT or MRI scan. Up-to-date high-end ultrasound devices were applied (Siemens Ultrasound Sequioa, ACUSON Sequoia, Mountain View, CA, USA, GE Healthcare E9, Chicago, IL, USA, Philips EPIQ7, Seattle, WA, USA) with proper CEUS protocols and at low mechanical index (<0.2) to prevent early destruction of microbubbles. For making appropriate fusion imaging feasible, an additional position sensor for the ultrasound probes and magnetic field generator (electromagnetic tracking) were required to allow for spatial tracking. First, the import of Digital Imaging and Communications in Medicine (DICOM) datasets from prior CT or MRI scans to the ultrasound devices was necessary before a thorough planning phase. Image matching can either be conducted in an overlaid or side-by-side and real-time manner, showing the same planes simultaneously. 

For CEUS, the intravenous administration of 1.0–2.4 mL of *SonoVue^®^*, a second-generation blood pool contrast agent (*Bracco*, Milan, Italy), and an additional 5.0–10.0 mL sterile 0.9% sodium chloride was done. No adverse side effects were observed upon administration of *SonoVue^®^*. Hepatic lesions were intermittently evaluated during the early arterial phase (10–45 s after i.v. application of *SonoVue^®^*), portal venous phase (30–120 s), late venous phase (2–6 min) for at least 5 min. Renal lesions were intermittently assessed during the cortical phase (8–35 s after intravenous application of *SonoVue^®^*), corticomedullary phase (36–120 s), and late phase (>2 min). Archived cine-loops of all included patients were retrospectively analyzed. All patient data and imaging files were stored and retrieved from the institutional picture archiving and communication system (PACS). 

Fusion imaging was technically successful in all included 92 patients. 

Renal or hepatic surgery was conducted in the local Department of Urology or in the Department of Surgery, respectively. The histopathological analysis was performed by the local Institute of Pathology. 

## 5. Conclusions

To our knowledge, this retrospective study encompassed the largest number of included patients on whom fusion imaging was performed for assessing renal lesions. Our findings highlighted the beneficial combination of real-time CEUS and cross-sectional imaging (CT/MRI) for evaluating hepatic and renal lesions. Its excellent safety profile, its accessibility and repeatability, its applicability in oncologic patients with kidney or thyroid affections, as well as in pregnant and young patients, and its cost-effectiveness are assets of fusion imaging, which, therefore, should be integrated into the daily routine of the modern radiologist.

## Figures and Tables

**Table 1:** Overview of findings from cross-sectional imaging (computed tomography/magnetic resonance imaging (CT/MRI)) and corresponding correlates by fusion imaging of 32 patients with focal liver lesions, CT-computed tomography, MR-magnetic resonance imaging.

CT/MRI	Fusion Imaging
Indeterminate lesions (*n* = 11)	Uncomplicated liver cysts (*n* = 4, 36%) Hemangioma (*n* = 3, 27%) Focal nodular hyperplasia (FNH) (*n* = 2, 18%) Hemorrhagic liver cyst (*n* = 1, 9%) Vascular pseudolesion (*n* = 1, 9%)
Suspicious lesions (*n* = 14)	Malignant lesions (*n* = 5, 36%) Hepatocellular carcinoma (HCC, *n* = 1) Cholangiocellular carcinoma (CCC, *n* = 1) Rectal cancer metastasis (*n* = 1) Breast cancer metastasis (*n* = 1) Hemangioma (*n* = 4, 29%) Focal nodular hyperplasia (FNH) (*n* = 2, 14%) Uncomplicated liver cyst (*n* = 1, 7%) No malignancy (*n* = 1, 7%) No specific correlation (*n* = 1, 7%)
Hemangioma (*n* = 2)	Hemangioma (*n* = 2, 100%)
Successful ablation of hepatic metastasis of ovarian carcinoma (*n* = 1)	Successful ablation of hepatic metastasis of ovarian carcinoma (*n* = 1, 100%)
Liver abscess (*n* = 1)	Liver abscess (*n* = 1, 100%)
Focal calcification (*n* = 1)	Focal calcification (*n* = 1, 100%)
Complicated liver cysts (*n* = 2)	Uncomplicated cyst (*n* = 1, 50%) Hemorrhagic liver cyst (*n* = 1, 50%)

**Table 2:** Overview of findings from cross-sectional imaging (CT/MRI) and corresponding correlates by fusion imaging in 60 patients with focal renal lesions, CT- computed tomography, MRI-magnetic resonance imaging.

CT/MRI	Fusion Imaging
Indeterminate lesions (*n* = 20)	Bosniak 1 lesions (*n* = 6, 30%) Bosniak 2 lesion (*n* = 1, 5%) Bosniak 2F lesions (*n* = 8, 40%) Bosniak 3 lesion (*n* = 1, 5%) Angiomyolipoma (*n* = 1, 5%) Renal infarction (*n* = 1, 5%) Pyelonephritis (*n* = 1, 5%) Indeterminate, benign lesion (histopathologically confirmed as angiomyolipoma) (*n* = 1, 5%)
Suspicious lesions (*n* = 30)	Bosniak 1 lesions (13%) Bosniak 2 lesions (*n* = 3, 10%) Bosniak 2F lesions (*n* = 4, 13%) Bosniak 3 lesions (*n* = 6, 20%) Bosniak 4 lesions (*n* = 13, 43%) Angiomyolipoma (*n* = 1, 3%) No specific correlation (*n* = 1, 3%)
Uncomplicated renal cysts (*n* = 3)	Bosniak 1 lesion (*n* = 1, 33%) Bosniak 2F lesion (*n* = 1, 33%) Bosniak 3 lesion (*n* = 1, 33%)
Angiomyolipoma	Angiomyolipoma (*n* = 1, 100%)
Hemorrhagic renal cysts	Bosniak 1 lesion (*n* = 1, 50%) Bosniak 2F lesion (*n* = 1, 50%)
Renal infarction	Renal infarction (*n* = 1, 100%)
Hematoma	Hematoma (*n* = 1, 100%)
Parenchymal defect after Cyberknife treatment	Parenchymal defect after Cyberknife treatment (*n* = 1, 100%)
Recurrence of clear-cell renal cell carcinoma (RCC) (*n* = 1)	Recurrence of clear-cell renal cell carcinoma (RCC) (*n* = 1, 100%)

## Supplementary Materials

The following are available online at https://www.mdpi.com/2072-6694/12/10/2821/s1, Table S1: Included patients with focal liver lesions who underwent CT-/MRI-CEUS Fusion Imaging. B—Native B-mode, CD—Color Doppler, CEUS—Contrast-enhanced ultrasound, CE—Contrast-enhancement, US—Ultrasound, CT—computed tomography, FNH—focal nodular hyperplasia, FU—Follow-Up, HCC—hepatocellular carcinoma. MRI—magnetic resonance imaging, RFA—radiofrequency ablation. Table S2: Included patients with renal lesions who underwent CT-/MRI-CEUS Fusion Imaging. B—Native B-mode, CD—Color Doppler, CEUS—Contrast-enhanced ultrasound, CE—Contrast-enhancement, US—Ultrasound, CT—Computed tomography, FU—Follow-Up, MRI—magnetic resonance imaging, RFA—radiofrequency ablation, RCC—Renal-cell carcinoma.
