# Supplementary material for: Advanced Fusion Imaging and Contrast-Enhanced Imaging (CT/MRI–CEUS) in Oncology
Source: Cancers (Basel). 2020 Sep 30;12(10):2821. doi: 10.3390/cancers12102821 (PMC7600560; doi:10.3390/cancers12102821)
Supplement: Supplementary file 1 [file cancers-12-02821-s001.pdf]

cancers

Supplementary materials: Advanced Fusion Imaging and Contrast-Enhanced Imaging (CT/MRI–CEUS) in Oncology.

Vincent Schwarze, Johannes Rübenthaler, Constantin Marschner, Matthias Philipp Fabritius, Johannes Rückel, Nicola Fink, Daniel Puhr-Westerheide, Eva Gresser, Matthias Frank Froelich, Moritz Ludwig Schnitzer, Nils Große Hokamp, Saif Afat, Michael Staehler, Thomas Geyer and Dirk-André Clevert

Supplemental Table S1. Included patients with focal liver lesions who underwent CT-/MRI-CEUS Fusion Imaging. B - Native B-mode, CD - Color Doppler, CEUS - Contrast-enhanced ultrasound, CE - Contrast-enhancement, US – Ultrasound, CT – computed tomography, FNH – focal nodular hyperplasia, FU – Follow-Up, HCC – hepatocellular carcinoma. MRI – magnetic resonance imaging, RFA – radiofrequency ablation.

Patient Number	Age	Sex	Size (cm)	Liver Segment	CT findings	MRI findings	Native B-mode, Color Doppler, CEUS	Fusion Imaging	Treatment / FU	Histopathology
1	67	F	1.6	4B	-	Indeterminate	B/CD/CEUS: No specific correlation	**Vascular pseudolesion**	-	-
2	81	F	4.5	Multiple	-	Indeterminate cystic lesions	B: hypoechoic<br>CD: -<br>CEUS: -	**Uncomplicated cystic lesions**	-	-
3	46	F	0.7	8	-	Indeterminate, hypervascularized lesion	No correlation due to steatosis hepatis	B: Ill-defined<br>CD: No hypervascularization,<br>CEUS: Central-to-peripheral CE<br>→ **FNH**	-	-
4	17	F	0,7	7	-	Suspicious lesion	B: isoechoic<br>CD: -<br>CEUS: -	**Uncomplicated cystic lesion**	-	-
5	27	F	1.0	2	-	Suspicious lesion	No specific correlation	B: Ill-defined, inhomogeneous, hypoechoic,<br>CD: -<br>CEUS: -<br>→ **No malignancy**	Segmental liver resection II/III	No malignancy
6	80	M	3.5	4/8	-	Complicated cyst	B: inhomogeneous, predominantly hypoechoic<br>CD: -<br>CEUS: -	**Hemorrhagic liver cyst**	-	-
7	51	F	0.9	5	Indeterminate	-	B: hyperechoic<br>CD: -<br>CEUS: perinodular CE, centripetal filling	**Hemangioma**	-	-
8	70	M	0.8	2	Indeterminate	-	B: hypoechoic<br>CD: -<br>CEUS: -	**Uncomplicated liver cyst**		
9	48	F	0.8	7	-	Suspicious lesion	Massive steatosis hepatis	**No correlation**		

							B/CD/CEUS: No specific correlation			
10	38	F	0.5	7	Indeterminate	-	B: well-defined, hypoechoic,<br>CD: -<br>CEUS: -	**Uncomplicated liver cyst**		
11	83	F	0.6	8	Indeterminate	-	B: inhomogeneous, predominantly hypoechoic,<br>CD: -<br>CEUS: -	**Hemorrhagic liver cyst**	-	-
12	66	M	1.5	6	Suspicious lesion	-	B: hypoechoic<br>CD: -<br>CEUS: early arterial CE, wash-out	**Liver metastasis**	Right hemihepatectomy	Metastasis (of rectal carcinoma)
13	64	M	1.5	6	Suspicious lesion	-	B: hyperechoic,<br>CD: -<br>CEUS: perinodular CE, centripetal filling	**Hemangioma**	-	-
14	71	F	1.5	7	Calcification	-	B: hyperechoic,<br>CD: twinkling<br>CEUS: -	**Focal calcification**	-	-
15	58	F	3.7	2/3	-	Suspicious lesion	B: inhomogeneous, predominantly hypoechoic,<br>CD: -<br>CEUS: early arterial CE; wash-out	**Primary liver tumor or metastasis**	Left hemihepatectomy	Cholangiocellular carcinoma
16	53	F	1.0	7	Suspicious lesion	-	Massive steatosis hepatis;<br>no specific correlation in conventional US	B: Hypoechoic,<br>CD: -<br>CEUS: perinodular CE, centripetal filling<br>→ **Hemangioma**	-	-
17	53	M	4.0	7	-	Encapsulated abscess	B: hypoechoic<br>CD: -<br>CEUS: peripheral CE	**Residual liver abscess**	-	-
18	65	F	0.5	4	-	Complicated cyst	B: hypoechoic,<br>CD: -<br>CEUS: -	**Uncomplicated liver cyst**	-	-
19	54	M	0.7	2	Suspicious lesion	-	B: hyperechoic,<br>CD: -	**Hemangioma**	-	-

							CEUS: perinodular CE, centripetal filling			
20	25	F	0.5	8	Indeterminate	-	No specific correlation	B: Hypoechoic, CD: - CEUS: - → **Uncomplicated liver cyst**	-	-
21	54	F	5.0	7	RFA	-	B: well-defined, hypoechoic CD: - CEUS: -	**Successful ablation of hepatic metastasis of ovarian carcinoma**	-	-
22	58	F	1.5	5	-	Suspicious lesion	Massive steatosis hepatis; no specific correlation in conventional US	B: Ill-defined, CD: - CEUS: perinodular CE, centripetal filling → **Hemangioma**	-	-
23	35	F	4.5	7	-	Suspicious lesion	B: hyperechoic CD: -, CEUS: Central-to-peripheral CE → FNH	**FNH**	-	-
24	39	F	1.8	8	Hemangioma	-	B: hyperechoic, CD: - CEUS: perinodular CE, centripetal filling	**Hemangioma**	-	-
25	43	M	6.2	5	Hemangioma	-	B: hyperechoic, CD: - CEUS: perinodular CE, centripetal filling	**Hemangioma**	-	-
26	43	F	< 1,0	Multiple	Suspicious lesion	-	B: hypoechoic CD: - CEUS: early arterial CE, wash-out	**Metastases**	Biopsy	Metastases (breast cancer)
27	65	M	2.5	2/4A	-	Suspicious lesion	B: hypoechoic, CD: -, CEUS: early arterial CE, wash-out	**HCC**	Left hemihepatectomy	HCC
28	20	F	1.7	4B	Indeterminate	-	B : hyperechoic, CD: - CEUS: perinodular CE, centripetal filling	**Hemangioma**	-	-
29	57	F	1.5	6	Indeterminate	-	B : hyperechoic, CD: -	**Hemangioma**	-	-

							CEUS: perinodular CE, centripetal filling			
30	46	F	3.4	6	-	Indeterminate	B: hyperechoic<br>CD: -,<br>CEUS: Central-to-peripheral CE → FNH	**FNH**	-	-
31	76	M	1.0	7	-	Suspicious lesion	B: hypoechoic<br>CD: -<br>CEUS: early arterial CE, wash-out	**HCC**	-	-
32	59	F	1.2	7	-	Suspicious lesion	B: hyperechoic<br>CD: -,<br>CEUS: Central-to-peripheral CE → FNH	**FNH**	-	-

Supplemental Table S2. Included patients with renal lesions who underwent CT-/MRI-CEUS Fusion Imaging. B - Native B-mode, CD - Color Doppler, CEUS - Contrast-enhanced ultrasound, CE - Contrast-enhancement, US – Ultrasound, CT – computed tomography, FU – Follow-Up, MRI – magnetic resonance imaging, RFA – radiofrequency ablation, RCC – Renal-cell carcinoma.

Patient Number	Age	Sex	Size (cm)	Localization	CT findings	MRI findings	Native B-mode, Color Doppler, CEUS	Fusion Imaging	Treatment / Follow-Up	Histopathology
1	79	F	2.5	R	Suspicious lesion	-	B: hyperechoic,<br>CD: -<br>CEUS: -	**Bosniak 1**	-	-
2	80	M	2.0	R	Suspicious Lesion	-	B: inhomogeneous, partially hyperechoic<br>CD: -<br>CEUS: early arterial CE, wash-out	**Bosniak 3**	Right Nephrectomy	Oncocytoma
3	76	M	2.0	R	-	Suspicious lesion	B: isoechoic,<br>CD: -<br>CEUS: early arterial CE wash-out	**Bosniak 4**	Right Nephrectomy	Chromophobe RCC
4	73	M	0.7	R	Suspicious lesion	-	B: septated, hypoechoic<br>CD: -<br>CEUS: -	**Bosniak 2F**	-	-
5	70	M	1.5	L	Uncomplicated cyst	-	B: hypoechoic,<br>CD: -	**Bosniak 1**	-	-

							CEUS: -			
6	53	F	3.0	R	Suspicious lesion	-	B: hypoechoic,<br>CD: -<br>CEUS: early arterial CE	**Bosniak 4**	-	-
7	44	M	1.0	L	Suspicious lesion	-	No specific correlation	**No specific correlation**	-	-
8	67	M	2.0	L	Indeterminate	-	B: hypoechoic,<br>CD: -<br>CEUS: -	**Bosniak 1**	-	-
9	48	F	0.5	L	-	Indeterminate	B: hypoechoic,<br>CD: -<br>CEUS: early arterial enhancement	**Indeterminate, benign**	Nephrectomy	Angiomyolipoma
10	72	M	1.2	L	-	Indeterminate	No specific correlation	B/CD: No specific correlation, CEUS: arterial CE<br>→ **Bosniak 3**	No treatment due to comorbidities	-
11	78	M	1.3	R	Suspicious lesion	-	B: hypoechoic,<br>CD: -<br>CEUS: -	**Bosniak 1**	-	-
12	60	M	1.5	R	Suspicious lesion	-	B: hypoechoic,<br>CD: -<br>CEUS: slight arterial CE	**Bosniak 3**	Right nephrectomy	Clear-cell RCC
13	75	M	1.2	L	Hemorrhagic renal cyst	-	B: hypoechoic,<br>CD: -<br>CEUS: septal arterial CE	**Bosniak 2F**	-	-
14	61	M	2.5	L	Suspicious lesion	-	B: inhomogeneous, hyperechoic,<br>CD: -<br>CEUS: early arterial CE, wash-out	**Bosniak 4**	Left nephrectomy	Clear-cell RCC
15	66	F	0.7	L	Suspicious Lesion	-	B: hypoechoic, multiple septations,<br>CD: -<br>CEUS: septal + nodular arterial CE	**Bosniak 4**	Left nephrectomy	Clear-cell RCC
16	70	M	1.0	L	Suspicious lesion	-	No specific correlation	B: hypoechoic,<br>CD: -<br>CEUS: -	-	-

								**→ Bosniak 1**		
17	47	F	3.5	L	-	Suspicious lesion	B: hyperechoic, CD: - CEUS: arterial CE	**Angiomyolipoma**	-	-
18	72	M	5.3	L	Hematoma	-	B: inhomogeneous, hyperechoic perirenal area CD: - CEUS: -	**No active bleeding, renal hematoma due to derailed oral anticoagulation (warfarin)**	-	-
19	80	M	3.0	R	Suspicious lesion	-	B: hypoechoic, CD: - CEUS: arterial CE, wash-out	**Bosniak 4**	Right nephrectomy	Clear-cell RCC
20	51	F	1.0	R	Suspicious lesion	-	B: isoechoic, CD: - CEUS: early arterial CE, wash-out	**Bosniak 4**	Right nephrectomy	Clear-cell RCC
21	70	M	8.0	R	Indeterminate cystic lesion	-	B: hypoechoic, septations, CD: -, CEUS: -	**Bosniak 2F**	Constant FUs	-
22	83	M	3.0	R	Suspicious lesion	-	B: hypoechoic, CD: -, CEUS: -	**Bosniak 1**	-	-
23	58	M	3.0	R	Suspicious lesion	-	B: hypoechoic, septations, CD: -, CEUS: -	**Bosniak 2F**	Right partial nephrectomy (15 months later)	Papillary RCC
24	79	M	1.6	L	Tumor recurrence (after partial nephrectomy due to clear-cell RCC)	-	B: hypoechoic, inhomogeneous, CD: - CEUS: early arterial CE, wash-out	**Tumor recurrence (RCC)**	-	-
25	50	F	1.5	R	-	Parenchymal defect after Cyberknife treatment of clear-cell RCC	B: isoechoic, rounded, CD: - CEUS: -	**Parenchymal defect after Cyberknife treatment of clear-cell RCC**	-	-
26	58	F	2.0	R	Indeterminate	-	B: hypoechoic, CD: - CEUS: -	**Bosniak 1**	-	-
27	51	M	1.6	L	Renal infarction	-	B: hypoechoic, patchy	**Renal infarction**	-	-

							CD: - CEUS: -			
28	87	M	0.7	R	Indeterminate	-	B: hypoechoic, CD: - CEUS: -	**Bosniak 1**	-	-
29	56	M	1.8	L	Suspicious lesion	-	B: inhomogeneous, solid components, CD: -, CEUS: early arterial CE, wash-out	**Bosniak 4**	Left partial nephrectomy	Papillary RCC
30	53	M	3.4	L	-	Indeterminate	B: hypoechoic, septations, CD: - CEUS: marginal + septal CE	**Bosniak 2F**	Constant in long-term FUs	-
31	64	F	3.5	R	Indeterminate	-	B: hypoechoic, septations, CD: - CEUS: dim septal + CE	**Bosniak 2F**	Partial right nephrectomy	No malignancy
32	71	M	4.5	L	Indeterminate	-	B: hypoechoic, septations, CD: - CEUS: dim septal CE	**Bosniak 2F**	Constant long-term FUs	-
33	53	M	1.2	L	Indeterminate	-	B: inhomogeneous, partially hyperechoic, CD: - CEUS: -	**Bosniak 2F**	Constant long-term FUs	-
34	56	M	2.5	R	Indeterminate	-	B: hypoechoic, CD: - CEUS: -	**Bosniak 1**	-	-
35	73	F	1.0	R	Suspicious lesion	-	B: mainly hypoechoic, inhomogeneous, CD: - CEUS:	**Bosniak 2**	Right partial nephrectomy	Angiomyolipoma
36	54	M	3.0	L	-	Suspicious lesion	B: hypoechoic, inhomogeneous, mural thickening, CD: -	**Bosniak 3**	Nephrectomy	Chromophobe RCC

							CEUS: marginal + septal CE			
37	66	M	5.0	L	Suspicious lesion	-	B: hypoechoic, CD: - CEUS: marginal + nodular CE	**Bosniak 4**	Nephrectomy	Papillary RCC
38	62	M	1.6	R	Hemorrhagic renal cyst	-	B: hypoechoic, CD: - CEUS: -	**Bosniak 1**	-	-
39	64	F	1.8	L	Uncomplicated renal cyst	-	B: inhomogeneous, mainly hypoechoic, CD: - CEUS: arterial central CE	**Bosniak 3**	-	-
40	61	M	1.5	R	Indeterminate	-	B: hyperechoic, CD: - CEUS: early arterial CE	**Inflamed morphology in the context of pyelonephritis**	Nephrectomy	No malignancy, pyelonephritis
41	68	F	2.0	R	Suspicious lesion	-	B: hypoechoic, CD: - CEUS: early arterial CE	**Bosniak 3**	No FU (palliative)	-
42	70	F	1.1	R	Indeterminate	-	B: hypoechoic, septations, CD: - CEUS: -	**Bosniak 2F**	-	-
43	68	F	7.5	L	-	Suspicious lesion	B: hypoechoic, septations, CD: - CEUS: dim septal CE	**Bosniak 2F**	-	-
44	68	F	1.7	L	Indeterminate	-	B: hypoechoic, septations CD: - CEUS: -	**Bosniak 2F**	Constant long-term FU	-
45	44	M	3.0	R	-	Indeterminate	B: hypoechoic, septations, CD: - CEUS: -	**Bosniak 2F**	-	-
46	68	M	0.8	L	Suspicious lesion	-	B: hypoechoic, inhomogeneous,	**Bosniak 2**	-	-

							CD: -<br>CEUS: -			
47	78	M	2.1	L	Indeterminate	-	B: hypoechoic, inhomogeneous,<br>CD: -<br>CEUS: -	**Bosniak 2**	-	-
48	67	F	3.5	L	Indeterminate renal cyst	-	B: hypoechoic,<br>CD: -<br>CEUS: -	**Bosniak 1**	-	-
49	80	M	5.0	R	Suspicious lesion	-	No specific correlation due to massive obesity	B: hypoechoic,<br>CD: -<br>CEUS: early arterial CE, wash-out<br>→ **Bosniak 4**	Right nephrectomy	Clear-cell RCC
50	69	F	3.5	L	Suspicious lesion	-	B: hypoechoic lesion,<br>CD: -<br>CEUS: early arterial CE	**Bosniak 4**	Left nephrectomy	Clear-cell RCC
51	75	F	1.0	R	Indeterminate	-	B: hyperechoic,<br>CD: -<br>CEUS: equivalent to renal parenchyma	**Angiomyolipoma**	Constant in long-term FU	-
52	47	M	2.0	R	Suspicious lesion	-	B: hyperechoic,<br>CD: -<br>CEUS: early arterial CE, wash-out	**Bosniak 4**	Right nephrectomy	Clear-cell RCC
53	68	M	10.0	R	Uncomplicated renal cyst	-	B: hypoechoic, septations,<br>CD: -<br>CEUS: -	**Bosniak 2F**	-	-
54	57	M	0.8	L	-	Indeterminate	B: hypoechoic,<br>CD: -<br>CEUS: -	**Bosniak 1**	-	-
55	71	M	1.0	L	Suspicious lesion	-	No specific correlation	B/CD: no specific correlation,<br>CEUS: early arterial CE, wash-out<br>→ **Bosniak 3**	Left nephrectomy	Oncocytoma
56	57	M	0.8	R	-	Suspicious lesion	No specific correlation	B/CD: no specific correlation,<br>CEUS: early arterial CE, wash-out	Right nephrectomy	Clear-cell RCC

								**→ Bosniak 4**		
57	62	M	2.0	R	-	Suspicious lesion	B: hypoechoic, septations<br>CD: -<br>CEUS: dim arterial septal CE	**Bosniak 3**	Right nephrectomy	Papillary RCC
58	53	M	1.3	R	-	Indeterminate	B: cortical retraction,<br>CD: -<br>CEUS: corresponding lack of CE	**Renal infarction**	-	-
59	31	F	1.4	L	-	Angiomyolipoma	B: hypoechoic,<br>CD: hypervascularized<br>CEUS: early arterial CE, wash-out	**Angiomyolipoma at 26th week of pregnancy**	Biopsy, constant during FUs	Angiomyolipoma
60	56	M	1.2	R	Suspicious lesion	-	B: hypoechoic,<br>CD: -<br>CEUS: early arterial CE, wash-out	**Bosniak 4**	Right partial nephrectomy	Clear-cell RCC

© 2020 by the authors. Licensee MDPI, Basel, Switzerland. This article is an open access article distributed under the terms and conditions of the Creative Commons Attribution (CC BY) license (http://creativecommons.org/licenses/by/4.0/).
